# The clinical utility of the IRAC component of the Framework for Routine Outcome Measurement in Liaison Psychiatry (FROM-LP)

**DOI:** 10.1192/bjb.2018.29

**Published:** 2018-08

**Authors:** Caroline Guest, Stephen Crockett, Patrick Little, Anish Patel

**Affiliations:** 1 Avon and Wiltshire Mental Health Partnership NHS Trust; 2 North Bristol NHS Trust, Bristol

## Abstract

**Aims and method:**

The clinical utility of the multidimensional Framework for Routine Outcome Measurement in Liaison Psychiatry (FROM-LP) has not previously been examined. We sought to establish whether referral accuracy and ability to achieve the reason(s) for referral to our liaison service improved after incorporating the Identify and Rate the Aim of the Contact (IRAC) scale of this tool into our referral process. We carried out a retrospective analysis of electronic case notes of all appropriate referrals to the team before and after this adaption.

**Results:**

Accuracy of referrals to our team improved from 73.8 to 93.7% following intervention. Referral requests that were fully achieved improved from 57.4 to 77.8%, and referral requests that were not achieved decreased from 26.2 to 6.4%.

**Clinical implications:**

The IRAC component of the FROM-LP measures what it was developed for, and thus has clinical utility supporting its widespread adoption across liaison services in the National Health Service.

**Declaration of interest:**

None.

The value and cost-effectiveness of an adequately funded and organised mental health liaison team (MHLT) have been demonstrated and promoted in national policy documents and economic analyses over the past 5 years.^1^^–^^4^ Although the economic benefits of a MHLT have been established, there is a lack of evidence relating to clinical (and other) outcomes.^5^ Measurement of clinical outcomes is essential for clinical teams to evaluate their work and enable ongoing service development. A Centre for Mental Health (CMH) report highlighted the challenges associated with measurement of outcome across MHLTs, which are operating in a number of different settings and carrying out a wide range of clinical activities in support of patients with many different types of clinical problems.^5^ A systematic review on the effectiveness of liaison psychiatry found that many studies had disparate results and were methodologically flawed.^6^ The CMH report proposed the use of a framework for measurements based on a logic model approach which took into account structure (inputs), process (activities) and outcomes (outputs) and suggested using a combination from each, ‘the so-called Scorecard approach’.^5^

## Development of the FROM-LP

In response to these recommendations, in 2015 the Faculty of Liaison Psychiatry of the Royal College of Psychiatrists produced the Framework for Routine Outcome Measurements in Liaison Psychiatry (FROM-LP).^7^ The authors, after further describing its development, proposed that it was adopted across all MHLT's in the National Health service (NHS).^8^ Their aim was to enable consistency of data collection and effective reporting of outcomes such that patients, referrers, the NHS Trust providing the service and commissioners could all understand and have confidence in the beneficial effects of their MHLT. The authors acknowledged that rolling out this tool quickly across MHLT's nationwide meant that it was potentially an imperfect tool; however, they recognised that it could be refined over time.^7^

## Implementation of the FROM-LP

Until recently, our MHLT had been measuring a number of outcomes; however, these were not based on national guidelines and therefore could not be directly compared with other MHLTs across the NHS. In addition, our in-patient team were assessing patients and finding that the reason for referral documented on the e-referral form was not always accurate. In April 2017, we adapted our routine data collection to include the FROM-LP outcome measurements. As shown in Table 1, there are three broad outcome categories. For the purposes of this evaluation, we focused on the *Process* section, which includes the ‘Identify and Rate the Aim of the Contact’ (IRAC) scale, comprising ten aims of contact (Table 1). We replaced the four referral options on the e-referral form with the ten IRAC categories (Table 2) and then evaluated whether this:

(a) improved the *accuracy* of reason for referral to our service
(b) in turn, improved our *ability* to fully achieve those reasons for referral, i.e. with this analysis could we determine the construct validity and thus the clinical utility of this component of the FROM-LP?

**Table 1:** Framework for Routine Outcome Measurement in Liaison Psychiatry (FROM-LP) content. Adapted from Trigwell & Kustow.^7^

FROM-LP summary table		
Measurement	Case type 1: single contact	Case type 2: series of contacts
Process	1. Response time **2. IRAC**	1. Response time **2. IRAC**
Outcomes (clinician-rated)	3. CGI-I	3. CGI-I (at beginning and end of series of contacts)
Outcomes (patient-rated)		4. CORE-10 (at beginning and end of series of contacts)
Patient satisfaction	4. Patient satisfaction scale 5. Friends and family test	5. Patient satisfaction scale 6. Friends and family test
Referrer satisfaction	6. Referrer satisfaction scale (as a regular survey if frequent referrers)	7. Referrer satisfaction scale (as a regular survey if frequent referrers)

CGI, Clinical Global Impression – Improvement scale; CORE-10, Clinical Outcomes in Routine Evaluation (10-item version); IRAC, Identify and Rate the Aim of the Contact.

**Table 2:** Adaption of the e-referral form incorporating the ten IRAC categories

Reason for referral categories of original e-referral form	IRAC (Identify and Rate the Aim of the Contact) categories incorporated into adapted e-referral form	Was it achieved?
1. Diagnosis	1. Assessment & diagnosis 2. Medication management 3. Assessment & management of risk	Fully achieved 2
2. Management of disturbed behaviour	4. Management of disturbed behaviour 5. Providing guidance and advice 6. Signposting & referring on	Partially achieved 1
3. Medication advice	7. Assessment of mental capacity 8. Mental Health Act 9. Brief psychological intervention	Not achieved 0
4. Capacity assessment	10. Treatments (other)	

It would also provide more detailed data about the type of activities our team were routinely carrying out.

## Method

Our MHLT is adult ageless and is based in a large 800-bed teaching hospital in Bristol. It is composed of doctors, nurses and social workers, and is split into an emergency department team and an in-patient team. This paper focuses on the in-patient, which received an average of 106 referrals per month during the period October 2016 to September 2017, often involving a series of patient contacts. The referrer has to complete an e-referral form which consists of a drop-down menu and free-text boxes. Once the referral form has been accepted and opened by administrative staff, it is then triaged by the shift coordinator. The clinical information provided by the referrer enables the shift coordinator to determine the urgency of the referral, and can also help facilitate decisions such as which member of staff might be most appropriate to see the patient.

The e-referral form includes a ‘reason for referral’ section, which has a drop-down menu from which the referrer can select more than one option. Table 2 shows the original e-referral form, which had four categories (left column), and the e-referral form adapted for our team by the Trust IT department, which has ten categories based on the IRAC scale. All the other information on the e-referral form remained unchanged.

Following the launch of the modified e-referral form, when a clinical member of our team closed a case, they were asked to record the reason for referral (categories 1–10, Table 2) and whether the reason for referral was fully achieved (2), partially achieved (1) or not achieved (0) in accordance with FROM-LP guidance. As the in-patient team did not collect these data prior to modification of the e-referral, our researcher (S.C.) rated whether the team had met the reason(s) for referral before and after the intervention so that a more direct comparison could be made.

### Data collection

Our researcher (S.C.) retrospectively reviewed the electronic healthcare records of patients referred to the in-patient team before and after the intervention. The initial group consisted of all appropriate referrals to our team from 3 to 16 Oct and 24 Oct to 6 Nov 2016. The comparison group consisted of all appropriate referrals to our service from 24 Apr to 7 May and 15 May to 28 May (2017). The electronic records were scrutinised for each patient referred to determine whether the reason for referral on the e-referral form was accurate (i.e. by probing the content of the assessments). If the reason for referral stated on the e-referral form was established as accurate, the researcher then further reviewed the electronic records to determine whether the in-patient MHLT had fully achieved, partially achieved or not achieved the reason(s) for referral. If the reason for referral on the e-referral form was not accurate, it was recorded as ‘did not achieve the reason(s) for referral’ (because it would not have been possible to meet the reason for referral if we had been given inaccurate referral information from the outset).

### Statistical analysis

The following outcomes before and after modification of the e-referral form were compared using Fisher's exact test:

(a) accuracy of the reason for referral
(b) referral outcome – did the in-patient MHLS fully achieve, partially achieve or not achieve the referral request?

The effect estimates are reported as odds ratios with 95% confidence intervals, and all the *P*-values reported are two-tailed.

## Results

A total of 124 cases were analysed; 61 were referred prior to the modification of the e-referral form and 63 were referred after. Comparison of the accuracy of the reason for referral before and after modification of the e-referral form (Table 3) demonstrated a statistically significant difference. Referrals were assessed as accurate in 73.8% of cases when using the previous referral system, compared with 93.7% when using the new referral system (*P* = 0.0030).

**Table 3:** Referral accuracy

Accurate	Before (*n* = 61)	After (*n* = 63)	Odds ratio (95% CI)	*P*-value
Yes, *n* (%)	45 (73.8)	59 (93.7)	5.24 (1.53–22.76)	0.0030
No, *n* (%)	16 (26.2)	4 (6.4)		

Comparison of whether the reason(s) for referral were met before and after modification of the e-referral (Table 4) also demonstrated a statistically significant difference. The referral request was assessed as fully achieved for 57.4% of referrals when using the previous referral system, and for 77.8% of referrals when using the new referral system (*P* = 0.0210). There were no significant differences identified between the two referral systems when the referral request was assessed as partially achieved (16.4 *v.* 15.9%, *P* = 1.0000). The percentage of referral requests assessed as not achieved decreased significantly when using the new system, from 26.2 to 6.4% (*P* = 0.0030).

**Table 4:** Referral outcomes

	Before (*n* = 61)	After (*n* = 63)	OR (95% CI)	*P*-value
Fully achieved, *n* (%)	35 (57.4)	49 (77.8)	2.60 (1.11–6.16)	0.0210
Partially achieved, *n* (%)	10 (16.4)	10 (15.9)	0.96 (0.33–2.82)	1.0000
Not achieved, *n* (%)	16 (26.2)	4 (6.4)	0.19 (0.04–0.65)	0.0030

## Discussion

The adapted e-referral form went live in March 2017. A retrospective analysis of the electronic healthcare records demonstrated that, following this intervention the reason, for referral was five times more likely to be accurate and the team was 2.6 times more likely to fully achieve the reasons for referral. The results suggest that improving the accuracy of the referrals improved the team's ability to achieve the reasons for referral.

Following the intervention, 6.4% of the referrals (compared with 26.2% before the intervention) were inaccurate. Periodic review of inaccurate referrals may help to determine why they were inaccurate. One possible explanation might be that the referrals to our team are generally made by the most junior doctor on the team, and the reason for referral may not have been made clear to them by the senior doctor asking for the referral. Another possibility is that none of the ten referral options adequately covered the reason for referral.

Following our intervention, the referral request was achieved (fully or partially) in 93.7% of referrals. In liaison work, it is not unexpected to partially achieve a referral request. Many patients might only be seen briefly prior to discharge from hospital, requiring handover to community teams or health professionals to complete the work. Despite this, further in-depth exploration as to the reasons would be useful.

An accurate referral to the MHLT is important because it enhances triage, so that patients can be prioritised accordingly and assigned to an appropriate member of staff (i.e. doctor or nurse, consultant or trainee doctor). It also potentially enables a more focused assessment based on the referrer's expectation; this could improve time efficiency, which in itself is important for a variety of reasons, such as when the patient is very unwell, or to facilitate financial savings and flow through the acute hospital. In addition, by outlining very specifically the referral categories to the referrers, it highlights exactly what type of work the MHLT can do and encourages referrers to consider the objectives of their referral, which in turn improves general efficiency.

We are aware that our evaluation, by embedding the IRAC scale into the referral form and asking the referrer to select the aim of contact, is in contrast to many other MHLT around the country, who tend to complete both the aim of the contact and achievement of the contact themselves. However, there were several reasons behind this process variance: (a) it was in alignment with the way many other specialties designed and operated their e-referral pathways in our trust, and so was not too dissimilar when making a referral to, e.g. respiratory or cardiology; (b) based on our experience, we felt that referrers often have a reasonably good idea of what they want assistance with in managing their patient, and it is then for us to be able to achieve that as providers of the service; and (c) it made data collection and measuring a much more reliable, more consistent and simpler process.

A number of limitations with this evaluation are worth commenting on. The number of patients involved in the retrospective analysis was small, and the confidence intervals were relatively wide as a consequence. A single researcher examined the electronic healthcare records for each referral, determining the accuracy of a referral based on the available clinical information and whether the reason for referral had been fully achieved, partially achieved or not achieved. This introduces the possibility of observer bias. If the researcher found the reason for referral to be inaccurate, then it was recorded as ‘not meeting the reason for referral’; this may have introduced exclusion bias.

In this evaluation, we did not measure or comment on referrer satisfaction, but we can predict that if there was an improvement in the team's ability to fully meet the reasons for referral, there would also be an associated improvement in referrer satisfaction. Using all the FROM-LP outcome measurements, our MHLT will be able to capture this information in the future. Our MHLT now routinely measures the IRACs for all referrals made to our service. Our results show that the main reasons for referral were:

(a) assessment & diagnosis (37.5%)
(b) medication management (11.8%)
(c) assessment and management of risk (12.5%)
(d) providing guidance and advice (16.5%).

This type of information can be used (alongside the structure and outcomes measurements in FROM-LP) to gain a clearer understanding of the work that the in-patient team are routinely carrying out, as described in the paper by Guthrie *et al*.^9^ This can then guide service development; for example, do our staff have all the necessary skills to manage the referrals, or do they require training in specific areas?

In the future, our team plan to incorporate the structure, process and outcomes data into a mental health dashboard on the Trust IT system, which will provide live up-to-date performance data, allowing our MHLT to anticipate trends quickly and respond in a timely fashion. This information will also be made available to our various ‘customers’ (commissioners, patients, carers, and referring staff).

### Conclusion

Since the launch of FROM-LP, there has been encouraging feedback based on opinion and observation, and numerous MHLT's have already implemented it.^8^ However, the developers acknowledged that rolling out this tool quickly across MHLTs nationwide meant that it was potentially imperfect.^7^ Tadros's commentary^10^ further encouraged MHLTs to develop a positive approach integrating the collection of outcome measures into everyday clinical practice, and found the FROM-LP a very useful tool to measure service quality and clinical effectiveness. To date, however, there has not been an actual appraisal of the tool or any part of it.

Through our evaluation of the IRAC scale of the FROM-LP, we have demonstrated an improvement in the accuracy of the referrals to our service. In turn, this has helped our team's ability to achieve the referral request and we have therefore been able to establish the instrument's construct validity. We conclude that the IRAC composition of FROM-LP does indeed measure what it was intended for, and we thus have demonstrated the clinical utility of the IRAC scale, which hopefully has reinforced its recommended incorporation into MHLTs across the NHS.

## About the authors

**Dr Caroline Guest** is a specialty doctor in Liaison Psychiatry in the North Bristol NHS Trust Mental Health Liaison Team, Bristol, UK; **Dr Stephen Crockett** and **Dr Patrick Little** are both Foundation Year 2 doctors in Liaison Psychiatry in the North Bristol NHS Trust Mental Health Liaison Team, Bristol, UK; and **Dr Anish Patel** is a Consultant Liaison Psychiatrist in the North Bristol NHS Trust Mental Health Liaison Team, Bristol, UK, and part of Avon and Wiltshire Mental Health Partnership NHS Trust, Chippenham, UK.
